# In-mold and Machine Sensing and Feature Extraction for Optimized IC-tray Manufacturing

**DOI:** 10.3390/polym11081348

**Published:** 2019-08-14

**Authors:** Shih-Chih Nian, Yung-Chih Fang, Ming-Shyan Huang

**Affiliations:** 1 Department of Power Mechanical Engineering, National Taitung Junior College, 911 Jhengci N. Rd., Taitung 95045, Taiwan; 2 Department of Mechatronics Engineering, National Kaohsiung University of Science and Technology, 1 University Rd., Yanchao, Kaohsiung City 824, Taiwan

**Keywords:** feature extraction, IC-tray, injection molding, parameter optimization

## Abstract

Injection molding is a mature technology that has been used for decades; factors including processed raw materials, molds and machines, and the processing parameters can cause significant changes in product quality. Traditionally, researchers have attempted to improve injection molding quality by controlling screw position, injection and packing pressures, and mold and barrel temperatures. However, even when high precision control is applied, the geometry of the molded part tends to vary between different shots. Therefore, further research is needed to properly understand the factors affecting the melt in each cycle so that more effective control strategies can be implemented. In the past, injection molding was a “black box”, so when based on statistical experimental methods, computer-aided simulations or operator experience, the setting of ideal process parameters was often time consuming and limited. Using advanced sensing technology, the understanding of the injection molding process is transformed into a “grey box” that reveals the physical information about the flow behavior of the molten resin in the cavity. Using the process parameter setting data provided by the machine, this study developed a scientific method for optimal parameter adjustment, analyzing and interpreting the injection speed, injection pressure, cavity pressure, and the profile of the injection screw position. In addition, the main parameters for each phase are determined separately, including injection speed/pressure during the mold filling phase, velocity-to-pressure switching point, packing pressure and time. In this study, the IC tray was taken as an example. The experimental results show that the method can effectively reduce the warpage of the IC-tray from 0.67 mm to 0.20 mm. In addition, the parameters profiles obtained by parameter optimization can be applied for continuous mass production and process monitoring.

## 1. Introduction

With its high manufacturing efficiency and low cost, injection molding is still one of the most commonly used processes in mass production today. Injection molding requires three basic elements: raw plastics, injection molds, and injection molding machines. For newly developed molds, molding tests are indispensable for confirming their effectiveness and the quality of the samples produced, which further affects the stability of continuous mass production. However, injection molding is a complex thermal-mechanical process the quality of which is greatly influenced by various factors: (1) product design, especially geometry and thickness; (2) mold design, especially gate location and type, runner, cooling channel, ejector, and venting; (3) injection molding machine, especially machine specifications, process parameter settings and machine stability; (4) auxiliary equipment; (5) variation in raw materials; (6) environment changes including temperature and humidity. Often, researchers try to improve the quality of injection molded parts by controlling screw position, injection and packing pressures, and mold and barrel temperatures. However, many studies have demonstrated that even with this control, the geometry of the molded part tends to vary significantly between different shots. Therefore, the precise control of the injection molding machine cannot successfully guarantee the quality of the injection molded parts. Further research is needed to properly determine the factors that affect the quality of the molten resin in each cycle, so that a more effective control strategy can be implemented [1].

**Figure 1:**
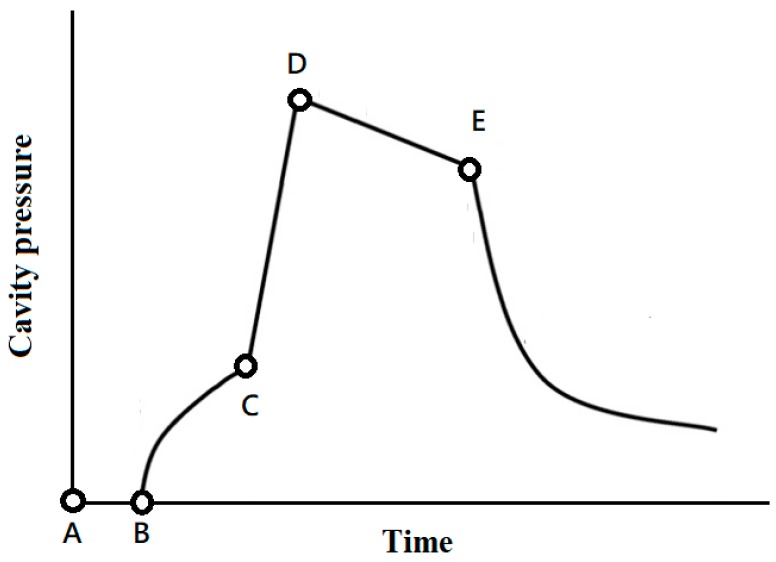
Typical cavity pressure profile.

Injection molding is a cyclic process consisting of four stages: filling, compressing, packing, and cooling/plasticizing. The cavity pressure has been proven to determine the repeatability of injection molding quality [2]. Figure 1 shows a typical cavity pressure curve in which the filling process begins at point A and then the cavity pressure signal begin at point B, where the molten resin initially contacts the pressure sensor, and then the pressure increases steadily as the filling progresses. The filling phase is completed at point C, where the cavity is volumetrically filled with molten resin without being compressed. The compression process then begins and the pressure quickly rises to the peak of point D. Thus, during the packing phase, as additional molten resin enters the cavity to compensate for shrinkage of the plastic, the melt within the cavity remains at the specified pressure. This process continues until the point at which the gate is sealed, as indicated by point E. The final cooling phase occurs after this and continues until the end of the cycle. During this phase, as the coolant circulating in the cooling channels in the mold removes heat, the molten resin gradually solidifies, with the rate of cooling and solidification determining the rate of decline of the cavity pressure.

Basically, the quality of injection molded parts is controlled by four main processing parameters, namely melt temperature, pressure, injection velocity and apparent viscosity. In particular, the viscosity consistency of the molten resin has a significant impact on the geometric accuracy and mechanical properties of the finished part. In fact, when the plastic enters the mold cavity, changes in the viscosity of the molten resin result in inconsistent flow behavior. This suggests that the varying pressure drops along the flow path may therefore result in part warpage [3,4]. Warpage control is generally considered to be the quality control of injection molding quality associated with continuous assembly quality and other issues. The main causes of warpage of injection molded parts is uneven volume shrinkage from high temperature to low temperatures. During the injection molding process, the pvT (pressure, specific volume and temperature) plots show the specific volume versus temperature and pressure dependence of the molten resin. The quality of the injection molded part is almost completely controlled by the pressure and temperature processing conditions.

Temperature is a key factor in the change of specific volume in processed plastics. Injection molded parts that are cooled from high temperatures have high shrinkage. When the temperature of the molten resin near the surface of the mold is lower than the temperature of the molten resin at the center, lower shrinkage results. Moreover, the thick portion shrinks more than the thin portion. Therefore, an injection molded part having a non-uniform thicknesses experiences inconsistent shrinkage and subsequent warpage. Differential cooling also results in changes in cross-sectional shrinkage. The temperature differences between the upper and lower part surfaces results in different shrinkage, resulting in warpage or residual stress after the part is ejected from the mold. Pressure also affects the specific volume of the plastic. For example, molten resin near the gate surrounded by high pressure shrinks less. Conversely, molten resin that is far from the gates and treated with low pressure shrink more. In the case of injection molding thin-walled parts, the molten resin is rapidly cooled, creating a pressure gradient in the thickness direction, resulting in uneven shrinkage after cooling. The direction of flow also affects shrinkage. The amount of shrinkage differs in a direction parallel and perpendicular to the direction of low orientation.

Although phenomena affecting the quality of the parts such as warpage have been well explained theoretically, in the past, injection molding was a black box for the operator due to the lack of physical information about the flow behavior of the molten resin in the cavity. Therefore, traditional process parameter settings are either based on statistical experimental methods, computer-aided simulations, or based on operator experience [5]. Such a process is time consuming and has limitations. If the settings for the production of injection molded parts are close to the specification limits of the part quality, the production process is easily affected by environmental noise and the defect rate increases. Therefore, these parameters are insufficient and the operation efficiency is not high. With advances in sensing technology, the so-called “black box” injection molding process has been transformed into a “grey box”’ by applying various sensors to reveal important information about the molten resin inside the machine and the mold. Therefore, this study developed an intelligent optimization parameter adjustment method to analyze the injection speed, injection pressure, cavity pressure, screw position, and process parameter setting data provided by the injection molding machine controller. The main parameters for each phase are individually adjusted, including injection speed/pressure during the mold filling phase, velocity-to-pressure switching time, and packing pressure and time. Through injection molding of an IC tray as an example of verification, this study proposes an initial result for the development of an intelligent calculation method that determines the above process parameter optimization.

## 2. Literature Review

Bozzelli and Grleau first mentioned scientific molding [6]. Kulkarni [7] provided scientific proof of why parts that meet quality requirement can (or cannot) be consistently molded. It is worth noting that scientific molding emphasizes that injection molding must change from “machine-centric” to “plastic perspective” [8], so the control of injection molding must evolve from traditional machine parameter control to melt state control. In scientific molding, it is essential to install the appropriate sensor in the right place to obtain important molding information. The application of various sensors in injection molding can be classified into three folds: (1) nozzle sensor, temperature or pressure sensors installed at the nozzle position to measure the melt statue in the nozzle; (2) in-mold sensor, temperature or pressure sensors installed in the mold to measure the state of the melt in the cavity during molding; (3) tie bar sensor, strain gages mounted on the surface of the tie bar to monitor the extension of the tie bar during clamping and injection. For example, Gornik [9] installed pressure and temperature sensors at different locations of the nozzle based on measurement data calculated from changes in melt viscosity during molding. For injection molding engaged with in-mold sensors, pressure and temperature sensors are typically installed inside the mold to monitor the melt state in the cavity [10,11]. Xie [12] developed a part weight control method using PT sensor and proved that cavity pressure is feasible as a reference for determining filling-to-packing switching point. In addition, the proposed PT packing control effectively improves the stability of the part weight. Moreover, Wang [13] also established a PT sensor to simultaneously track the melt pressure and temperature changes on the cavity surface during the molding process, and further control the shrinkage of the molded part according to the pvT theory. Gao and Gordon [14,15] applied a multivariate sensor (MVS) to online monitoring of part mass, dimensions, and structural characteristics. The MVS sensor detect pressure and temperature signals through piezo-ceramic elements and infrared thermopiles mounted in the sensor head. This information is further interpreted to obtain the flow rate at the melt front and the transient response of the infrared thermopile because the melt front flows through the lens of the sensor and then the apparent melt viscosity is obtained. Chen et al. [16] used a pressure sensor and an infrared temperature sensor to detect changes in pressure and temperature of the polymer melt during filling. He also proposed a pvT curve method to control the shrinkage and warpage of the molded parts. During the initial packing phase, the molten resin is compressed to fill the cavity with a high packing pressure that minimizes the specific volume at the end of the filling path, followed by dynamic pressure control to adjust the second and third stage pressures. The results show that the pvT curve method can efficiently improve the shrinkage change between the near gate and the end of filling, thereby reducing the warpage of the molded parts. In order to prevent sensor marks from appearing on the molded part, Lin [17] designed a pressure sensor bushing module near the sprue to monitor the change in injection pressure. Zhao [18] proposed the use of non-destructive ultrasonic sensors for melt pressure monitoring. Ultrasonic sensors can automatically identify different stages of the molding process without damaging the mold structure.

For the tie bar sensor technology, GEFRAN has introduced a tie-bar measurement system that reflects changes in melt pressure in the cavity during injection molding [19]. The test report shows that the measurement curve of tie-bar strain is consistent with the change of the melt pressure in the cavity. The tie-bar measurement system responds immediately to process changes and can be used as an indicator of the stability of the molding process. The tie-bar measurement system only needs to be installed on the machine and is suitable for different molds without making any changes to the structural design of the mold. Huang et al. used four linear variable differential transformers and four tie-bar strain sensors to study the relationship between molded part quality, tie-bar elongation, and mold separation in different molding processes [20]. The results show that different gate locations, cavity configurations, and injection pressures can be reflected in the tie-bar strain. It has been shown that the tie-bar strain curve can be used to monitor the overall variation of the molding process.

This study developed an intelligent molding test method that analyzes injection speed, injection pressure, cavity pressure, screw position, and process parameter setting data provided by the injection molding machine controller. The main parameters for each phase are individually adjusted, including injection speed/pressure during the mold filling phase, velocity-to-pressure switching point (V/P switching), packing pressure and packing time. Through the injection molding of the IC tray as an example of experimental verification, this study proposes an initial result for the development of an intelligent calculation method, which determines the above process parameter optimization.

## 3. Methodology

Table 1 shows the content of the intelligent mold testing technique, including two steps: (1) CAE simulation, and (2) actual molding. The CAE simulation step includes product design and mold design: the former mainly checks the product geometry, thickness distribution, insert and contact surface characteristics required in the mold design. It is worth noting that a proper thickness distribution can reduce problems that may occur during continuous injection molding. The latter evaluates with the flow balance primarily by the gate location, and the mold temperature distribution shows the performance of the cooling channel layout. These assessments are critical to ensure high quality injection molding based on proper mold design. The actual molding step consists of two stages: setting the parameters and obtaining a robust molding profile. The former determines the minimum injection speed to ensure a stable viscosity of the molten resin. The injection speed stage corresponds to the change in the cross-sectional area during mold filling, minimizing the packing time, and the packing pressure stage to achieve minimum warpage of the molded part.

**Figure 2:**
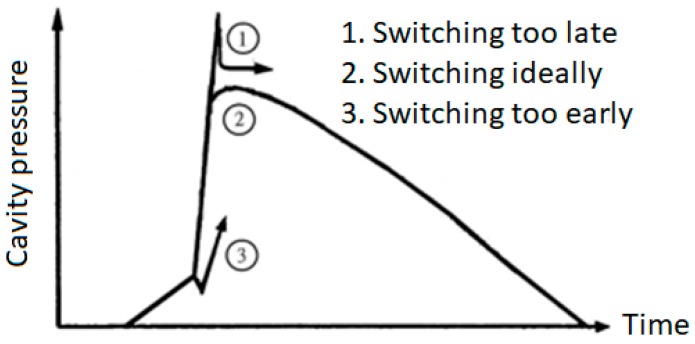
Three types of cavity pressure profiles correspond to switching times.

As mentioned before, the cavity pressure profile has proven to be one of the best indicators of the injection molding process, with which the characteristics of filling, compression, packing, and cooling qualities can be analyzed. For example, the transfer from the filling stage to the packing stage shows a sudden pressure drop followed by a rise; the overall packing rate and cooling rate can be observed along the slope, respectively. In particular, insufficient packing time results in a significant change in the pressure slope along the cooling stage. Therefore, the pressure profile is representative of the physical behaviors of the molten resin in the cavity and can then be used to determine whether to select the appropriate process parameters. Based on the information observed in the machine and the mold, the proposed automatic optimal parameter adjustment method is explained as follows:

(1) Optimizing the injection speed and pressure during the filling phase: The injection molding machine performs a speed control and an upper limit pressure strategy on the injection screw, which pushes the molten resin to fill the cavity during the filling phase. The first task is to check if the injection pressure is set high enough to provide enough force for the desired injection speed. In particular, the filling time value multiplied by the highest actual injection pressure with respect to various injection speeds reflects the viscosity characteristics. The next step is to optimize the injection speed to ensure minimal viscosity fluctuations, which yields the most stable flow quality of the molten resin.
(2) Optimizing velocity-to-pressure switching time: By observing the screw position and pressure history profile, the early, late or ideal switching time from the speed control of the injection screw to the pressure control during the mold filling and packing phase is determined. The first has a significant pressure drop and then rises at the switching point, the second has an apparent peak pressure, and the third has a relatively smooth pressure profile (Figure 2).
(3) Optimizing the packing pressure and time during the packing phase: By observing the screw position and hold time, a minimum packing time sufficient to compensate for the plastic shrinkage is determined. In addition, the optimum packing pressure is set to an averaged value of the lowest and the highest packing pressures which do not cause defective injection molding quality.

## 4. Experiments

In this study, a nozzle pressure sensor and three cavity pressure sensors (SSB01KN08 × 06, Futaba Co., Chiba, Japan) were used to detect changes in polymer melt pressure during injection molding. The pressure curves detected by the sensor are further used to achieve the following four objectives: (1) formulate rules for determining the V/P switching time, (2) establish gate frozen time rules, (3) pass pvT’s packing pressure adjustment to improve part shrinkage, (4) achieve a robust molding pressure profile that can be used as a standard profile for molds operating in different machines as well as quality monitoring in mass production.

**Figure 3:**
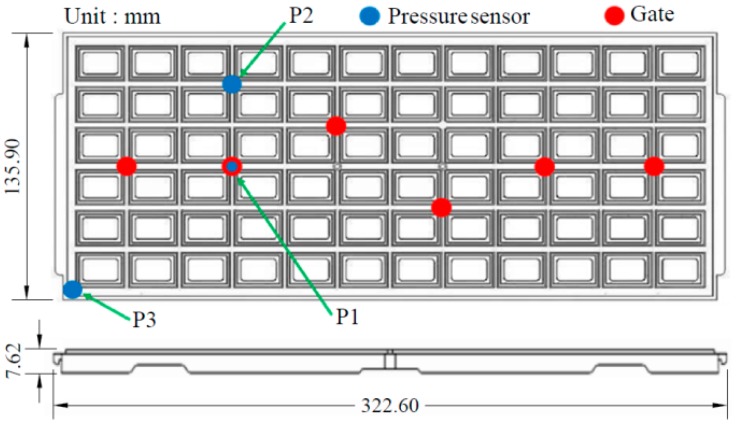
IC tray geometry and sensing location.

**Figure 4:**
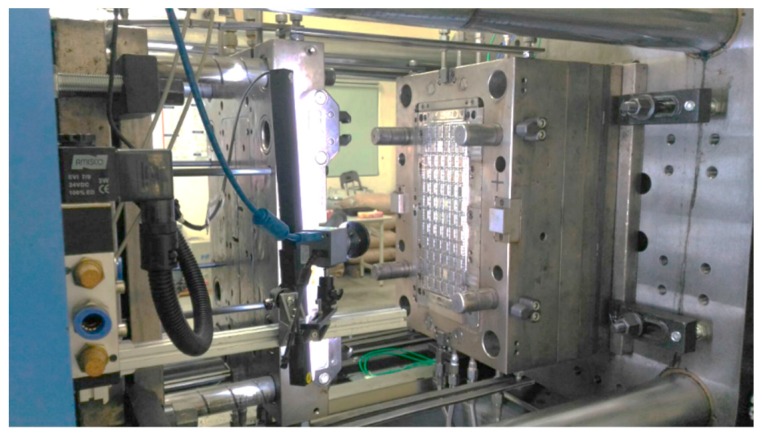
IC tray mold and injection molding machine.

**Figure 5:**
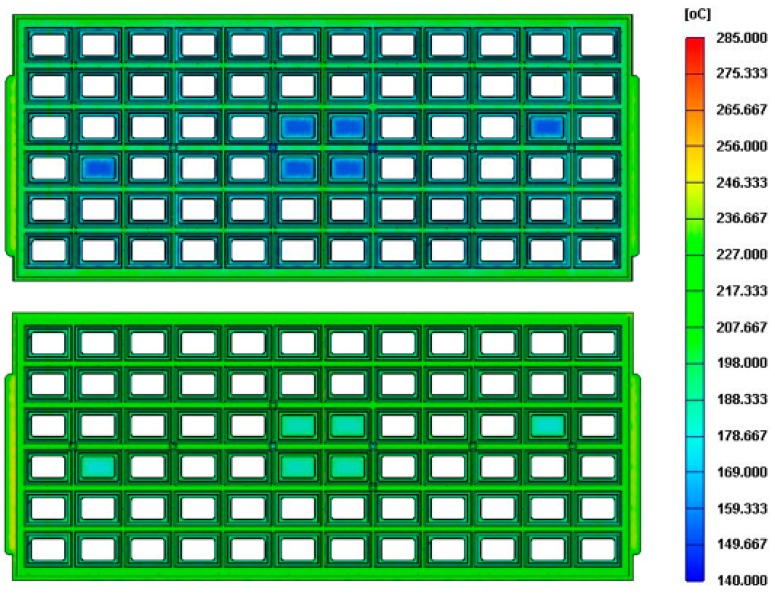
Temperature distribution of IC trays corresponding to different cooling channel designs: initial design (**top**) and improved design (**bottom**).

In this experiment, an IC-tray with a length of 322.6 mm and a width of 135.9 mm was molded by a domestic injection molding machine, which was an AH-200 hydraulic drive machine manufactured by Fu Chun Shin Machinery Manufacturing Co. (Tainan, Taiwan). The maximum clamping force is 200 tonnes, the maximum injection speed is 300 mm/s, and the screw diameter is 44 mm) Figure 3 shows the dimensions of the IC-Tray and the measurement position of the pressure sensor, with three pressure sensors installed close to, in the middle and distally from the gate, namely P1, P2, and P3. The plastic material applied was 35% glass fiber reinforced PPE. The warpage of the parts was measured by an accurate three coordinate measuring machine (CRYSTA-Apex S 7106 made by Mitutoyo Co., Kawasaki, Japan). Figure 4 shows the injection molding machine and IC tray mold used in the experiment. Since a good mold design is an essential element of the molding test, Figure 5 shows an example of a case study in which the temperature uniformity of the IC tray is greatly improved by modifying the initial cooling channel design through computer simulations of IC-tray association with Moldex3D commercial analysis software.

## 5. Results and Discussion

### 5.1. Injection Speed and Pressure Optimization during Filling Phase

First, the injection pressure is set high enough to provide sufficient force for the desired injection speed set by the injection molding machine that pushes molten resin to fill the cavity in the filling phase. Then, various injection speeds from 95% of machine specification to the lowest allowing 95% cavity volume filling are performed. The filling time value relative to the various injection speeds is multiplied by the highest actual injection pressure that reflects the viscosity characteristics. The results show that the ideal injection speed is 180 mm/s, allowing the lowest viscosity fluctuations, which yields the most consistent flow quality of molten resin.

### 5.2. Velocity-to-Pressure Switching Time Optimization

**Figure 6:**
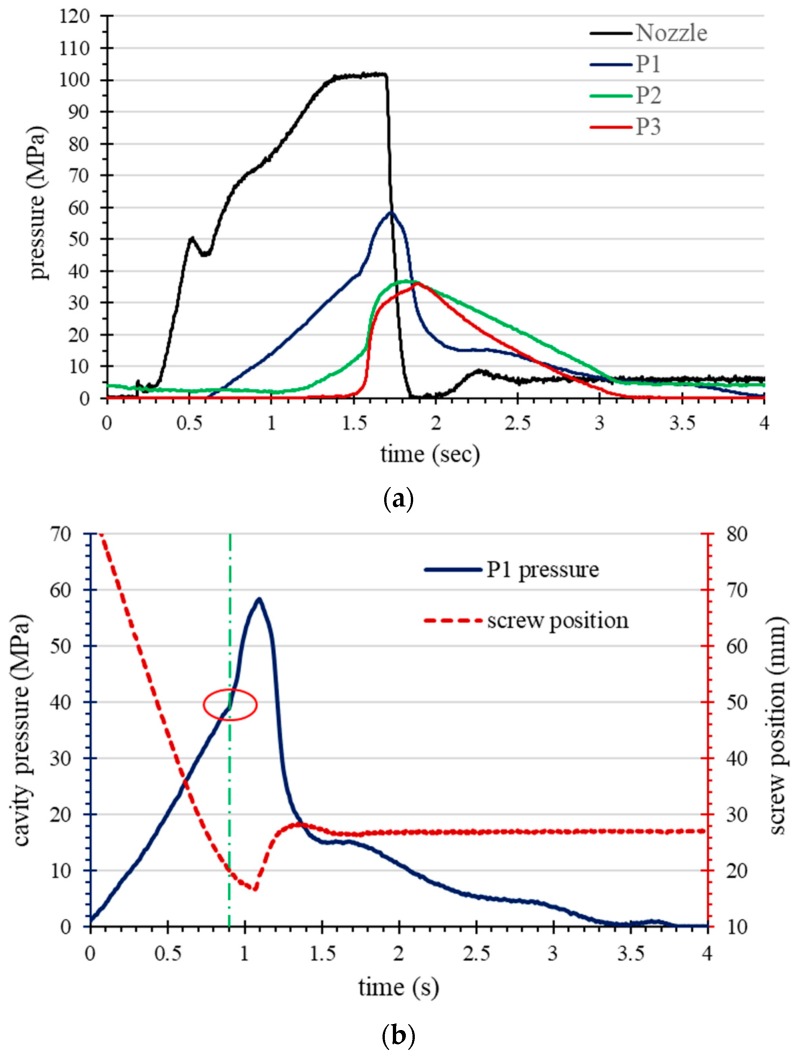
Cavity pressure profile and screw position with over-filling (no packing): (**a**) pressure profiles as the screw stroke acts on the fully filled cavity without packing; (**b**) the screw position of the completely filled cavity can be identified as about 17 mm.

**Figure 7:**
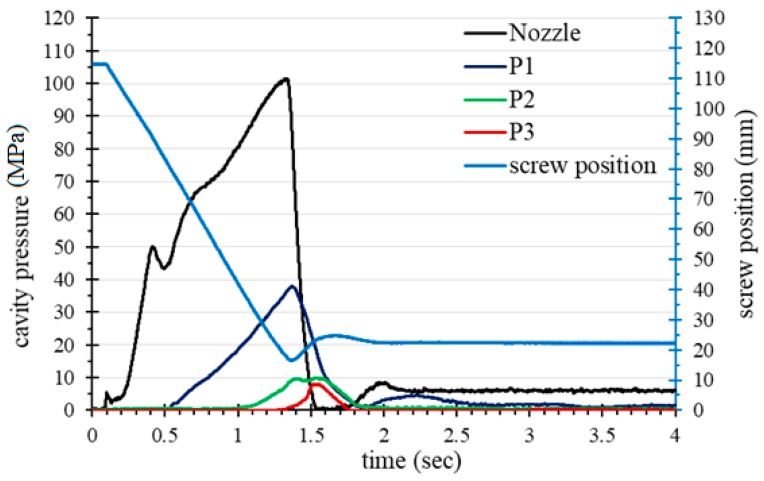
Cavity pressure profile and screw position with over-filling (no packing).

**Figure 8:**
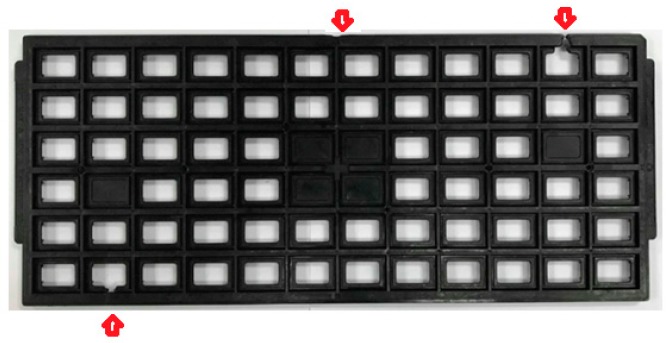
Short shot defects (without packing) for IC trays with under-filling.

This study developed a single screw position identification technique in which a cavity pressure profile is used to determine the screw position of a fully filled cavity. Table 2 shows the injection molding operating parameters and Figure 6a shows the pressure profile as the screw stroke acts on the fully filled cavity without packing. The pressure profiles show that when the cavity is completely filled, the behavior of the molten resin changes from flow to compression, which causes a sudden rise in cavity pressures (P1, P2, and P3). The point on the sudden raised pressure profile indicates the time at which the cavity is completely filled. Therefore, the screw position of the completely filled cavity can be identified as about 17 mm (see Figure 6b). The study showed that the appropriate switching time was set to about 95% volume filling (in this case about 17 mm in the screw position) to prevent over-packing. Moreover, Figure 7 shows cavity pressure profiles for changing the V/P switching, where the position is set to 95% of the unpacked screw stroke, and Figure 8 shows the corresponding part with short shot. It has been shown that when the cavity is not completely filled, the pressure profile does not have a rapidly increasing value.

### 5.3. Gate Freezing Time Experiment

The parts of the multiple packing stages in injection molding are set with the packing time. The so-called effective packing time is the duration from the starting of the V/P switching time to the gate freezing time. When the packing time is set shorter than the gate freezing time, the injection pressure at the end of the packing will be released. It further causes the molten resin to flow back from the cavity and can seriously affect the quality of the part. Conversely, long packing times result in wasted energy. Therefore, the gate freeze time should be identified first before setting the time for multiple packing stages.

Conventional mold testing determines the gate freezing time by measuring the changes in part weight as packing time is varied. It is worth noting that an effective and long packing time gradually increases the weight of the part. The minimum packing time, i.e., the gate freezing time, is the time during which the weight of the part remains stable as the packing time increases. This conventional method requires repeated operations during which the mold temperature, melt temperature, and packing pressure are changed. This study used a near-gate pressure profile (labeled P1) to quickly determine the gate freezing time with a single shot, which has the advantage of saving time and increasing precision.

**Figure 9:**
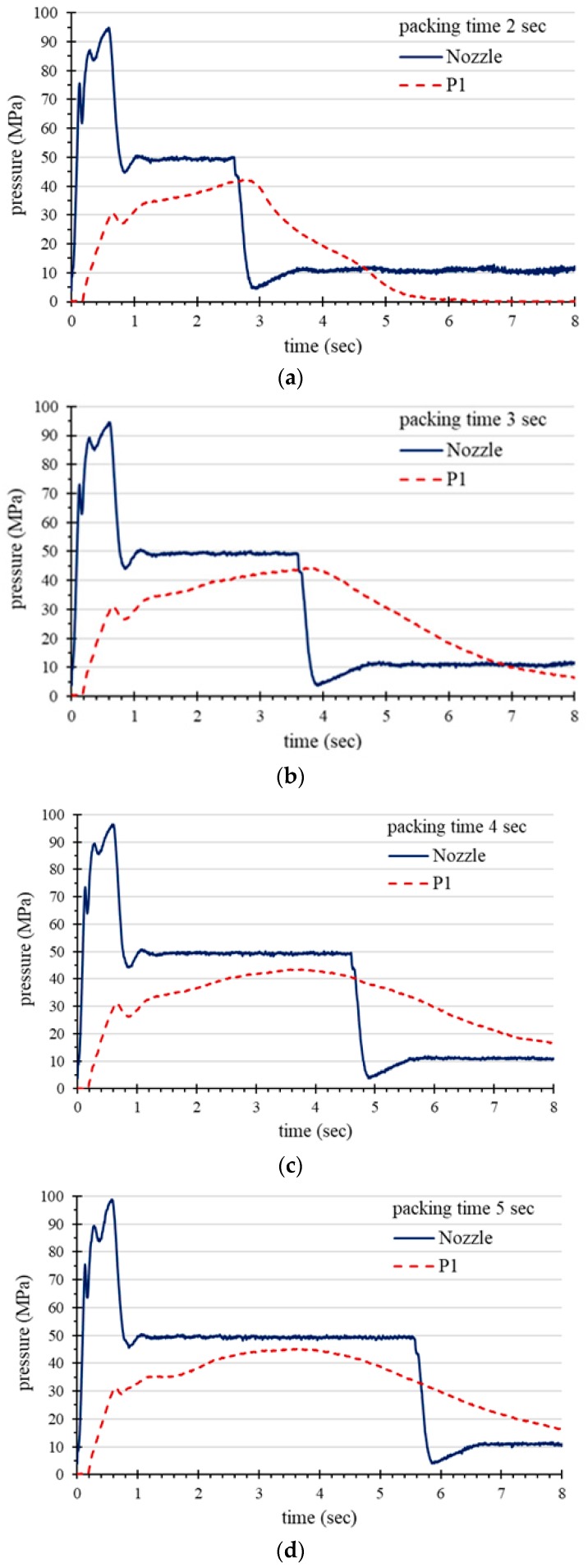
Pressure profiles w.r.t. various packing time: (**a**) 2 s, (**b**) 3 s, (**c**) 4 s, and (**d**) 5 s.

Figure 9 shows the pressure profiles of the near-gate sensor, i.e., P1 under different packing time. The results show that when the packing time is below the gate freezing time (Figure 9a,b), the P1 pressure profile will decrease at the end of packing time. However, when the packing time is longer than the gate freezing time (Figure 9c), the cavity pressure does not fluctuate when the packing pressure is released. It can be shown that when the packing time is longer than the gate freezing time, the P1 pressure profile does not decrease significantly at the end of the packing time. Figure 9d shows that when the packing time is longer than the gate freezing time, the increase in packing time does not affect the pressure profile of P1. Since the gate has been frozen, the molten resin pressure near the gate cannot be maintained by the packing pressure and the cavity pressure begins to decrease. Therefore, it is possible to apply a shot with a long packing time and a pressure profile detected near the gate as a feature for identifying the gate freezing time. Taking the IC-Tray molding as an example, the gate freezing time is about 3.8 s.

**Figure 10:**
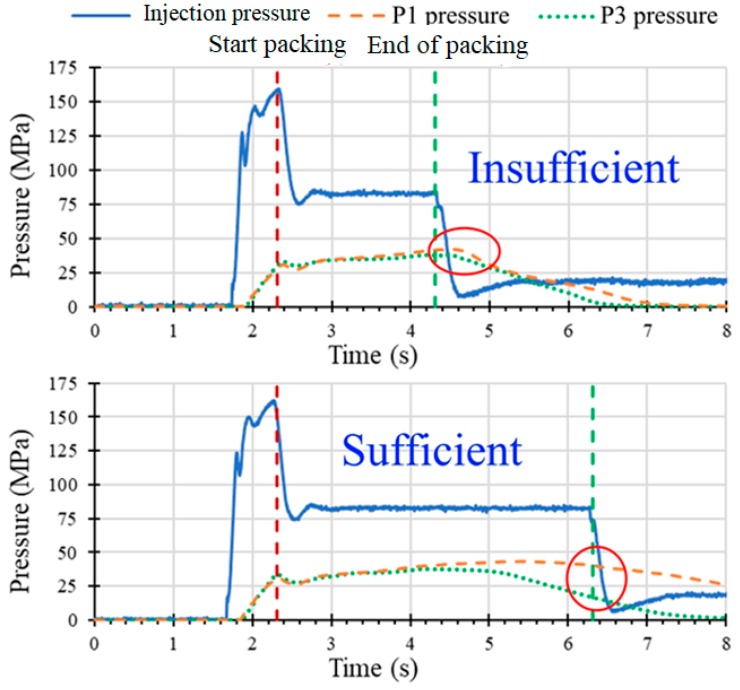
Determination of packing time: sufficient (**top**) and insufficient (**bottom**).

Figure 10 shows an example of determining the minimum packing time by observing the drop in the cavity pressure profile after the end of the packing.

For an insufficient packing time setting, the gate does not freeze, causing a portion of the molten resin to flow out of the cavity, thus causing a sharp drop in the cavity pressure profile immediately after the release of the packing pressure. Conversely, sufficient packing time maintains a relatively smooth reduction in the cavity pressure curvature.

### 5.4. Packing Pressure/Time Optimization in the Packing Phase

**Figure 11:**
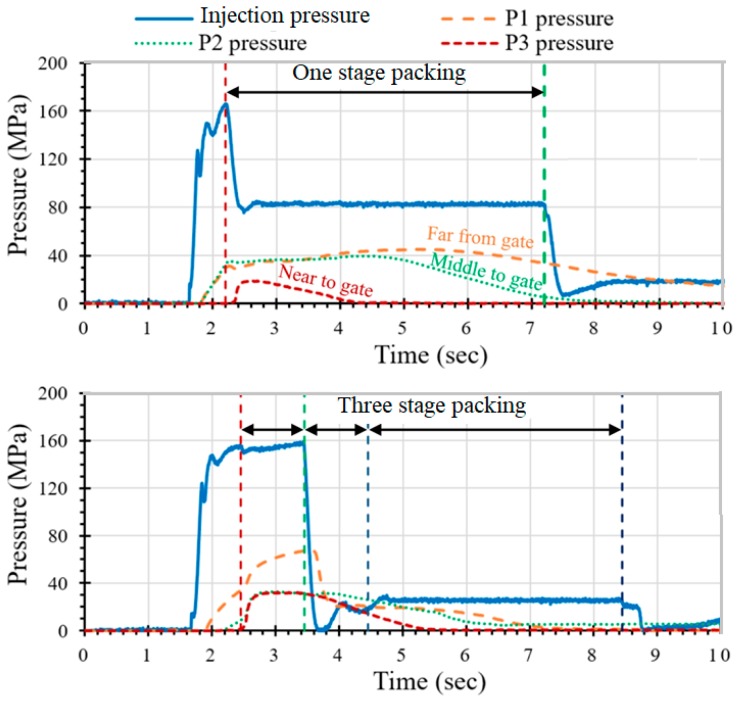
The pressure profile relative to: one-stage packing (warpage: 0.60 mm) (**top**); three-stage packing (warpage: 0.42 mm) (**bottom**).

Regarding the optimization of the packing pressure, multiple packing stages are often used to minimize the degree of warpage of an IC tray. The packing pressure profile is the most important factor affecting the shrinkage and warpage of the part. Figure 11(top) shows a pressure profile using single stage packing (pressure of 80 MPa). It can be observed that the pressure profile between the near-gate sensor P1 and the far-from-gate sensor P3 is completely different. When the P3 pressure drops to zero, the pressure of P1 still exceeds 20 MPa. According to the pvT theorem, the curves of P1, P2 and P3 should be closely reduced to minimize shrinkage. In this case, a large shrinkage change between the beginning and the end of filling path (i.e., sensors P1 and P3) will cause a large degree of warpage. The result also showed that warpage of the IC tray molded at the one-stage packing setting was 0.60 mm.

Figure 11(bottom) shows the pressure profile for the three-stage packing and Table 3 shows the process parameters setting. At the first stage of high pressure packing (160 MPa), the P1 and P2 pressures initially rise, then the P1 pressure is released when the second stage begins to pack. Finally, in the third packing stage, the injection pressure is maintained at 40 MPa. Through the pressure regulation of the three-stage packing, the pressure difference between P1 and P3 can be successfully reduced to within 2 MPa. On the contrary, with the three-stage packing setting in Figure 11(bottom), the change in shrinkage rate was reduced, and the warpage of the molded part was also substantially lowered to 0.42 mm. Therefore, this example successfully demonstrates the effect of optimizing packing pressure based on the sensor readings of the molten resin in the cavity.

### 5.5. Robust Molding Window Experiment

**Figure 12:**
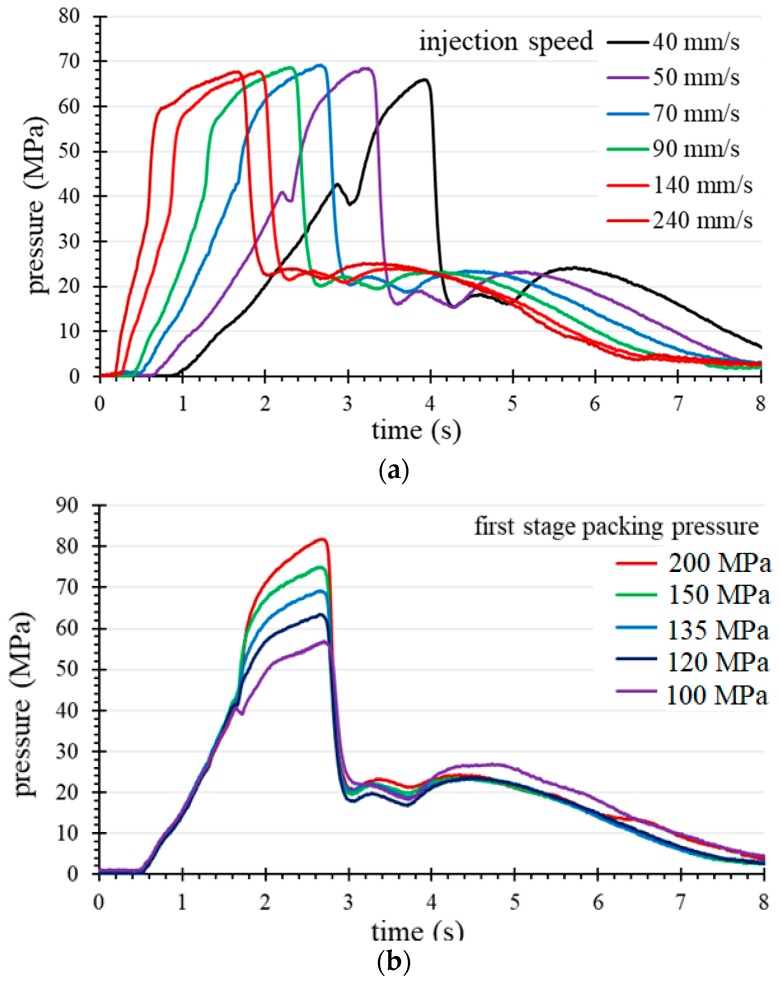
The P1 pressure variation with (**a**) injection speed (first stage packing pressure 135 MPa) and (**b**) first stage packing pressure (injection speed 70 mm/s).

The peak pressure of the cavity decisively affects the quality of the molded part. In this experiment, we attempted to disturb the pressure peak of P1 by adjusting the injection speed and pressure parameters to establish an operational window. For this part, the warpage tolerance of the molded parts is required to be 0.42 mm or less. By adjusting the injection speed and the packing pressure of the first-stage packing, the pressure peaks of the P1 curve are disturbed. Figure 12a,b respectively show the range of variation of the P1 pressure peak relative to the injection speed and the first stage packing pressure setting, which meets the requirements for warpage tolerance. Based on this range, a full factor experiment was performed to explore the relation between the pressure peak and the amount of warpage.

**Figure 13:**
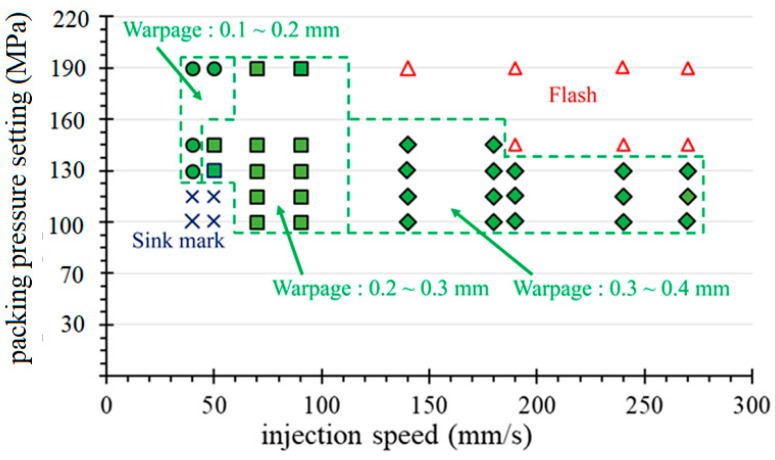
The warpage quality contour of the molded part corresponding to the injection speed and the packing pressure settings.

**Figure 14:**
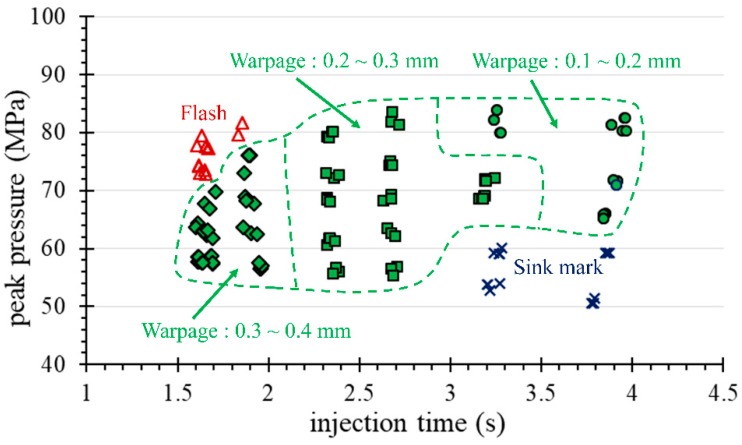
The warpage quality contour of the molded part corresponding to the P1 peak pressure and the injection time.

As for the goal of obtaining a robust molding profile [21], Figure 13 and Figure 14 show a comparison of the quality profiles with the machine set point (machine-centric) and quality index (plastic perspective) extracted from the process parameters. Figure 13 shows that the optimum setting for robust operation is at a packing pressure setting of 135 MPa and an injection speed of 70 mm/s. In comparison with Figure 13, the optimum parameters in Figure 14 for the first stage packing is a peak pressure of 70 MPa and an injection time of 2.8 s. These two numbers represent information about the process of obtaining robust parameters, but are completely different. Figure 13 shows a narrow process window for obtaining the minimum warpage (0.1 to 0.2 mm) of the IC tray corresponding to the set values of the packing pressure and the injection speed. Therefore, the ideal injection speed needs to be fine-tuned and can be easily omitted in a machine-centric situation. In contrast, the quality indicator domain in the plastic perspective shown in Figure 14 is more advantageous because it reveals its strict relationship to warpage, which can be used not only for quality monitoring, but also as a reference for quality control. In addition, the robustness parameter setting indicated by the quality indictor domain is independent of the operating injection molding machines in continuous production after molding test. This advantage facilitates fast and consistent quality manufacturing in injection molding plants.

## 6. Conclusions

The purpose of this study was to establish a standard intelligent molding test procedure based on process parameters and mold sensing signals to obtain a set of optimum process parameters, with molding test information further used as a reference and quality check for process parameter optimization. This research uses cavity pressure sensors to track the molten resin flow state during the injection molding process and adjust the molding parameters through the obtained pressure profiles. This work also established the decision rules for V/P switching time, gate freezing time, and multi-stage packing setting. Verification of IC-tray molded parts shows that the adjusted three-stage packing can achieve well-controlled part warpage (less than 0.42 mm) and no surface defects. In addition, the robust molding setup further minimizes part warpage to 0.20 mm or less. Also, a robust molding window containing peak pressure and injection time can be used for quality monitoring of part warpage. In summary, this work provides a preliminary study that allows injection molding engineers to systematically and scientifically manipulate process parameter settings without the need for time-consuming trial-and-error testing or with limited personal experience. In summary, the proposed method is believed to be helpful to increase the injection molding rate.

## Figures and Tables

**Table 1:** Intelligent mold testing technique.

CAE Simulation	Actual Molding
Product design stage: Geometry Thickness distribution Insert and contact surfaces	Process parameter setting: Minimum injection speed Injection speed stages Packing time Packing pressure stages
Mold development stage: Gate location Mold temperature distribution	Robust parameter setting: Quality window related to peak pressure and injection time

**Table 2:** V/P switching time optimized injection molding parameter settings.

Parameter (units)	Value
Injection pressure (MPa)	120
Injection speed (mm/s)	180
Packing pressure (MPa)	130
Melt temperature (°C)	330
Mold temperature (°C)	135

**Table 3:** Three-stage packing parameter settings.

Stage of Packing	Packing Pressure (MPa)	Packing Time (s)
1st	135	1
2nd	17	1
3rd	34	4

## References

[1] Chen J.Y., Yang K.J., Huang M.S. (2018). Online quality monitoring of molten resin in injection molding. Int. J. Heat Mass Transf..

[2] Huang M.S. (2007). Cavity pressure based grey prediction of the filling-to-packing switchover point for injection molding. J. Mater. Process. Technol..

[3] Nian S.C., Wu C.Y., Huang M.S. (2015). Warpage control of thin-walled injection molding using local mold temperatures. Int. Commun. Heat Mass.

[4] Nian S.C., Li M.H., Huang M.S. (2015). Warpage control of headlight lampshades fabricated using external gas-assisted injection molding. Int. J. Heat Mass Transf..

[5] Kazmer D., Barkan P. (1997). Multi-cavity pressure control in the filling and packing stages of the injection molding process. Polym. Eng. Sci..

[6] Brett L. Scientific Molding. https://www.scientificmolding.com.

[7] Kulkarni S. (2017). Robust Process Development and Scientific Molding: Theory and Practice.

[8] Doyle K. (2012). Top 10 Reasons Why Molders Fail at Implementing Scientific Molding. Plast. Technol..

[9] Gornik C. (2008). Viscosity measuring methods for feedstocks directly on injection molding machines. Mater. Sci. For..

[10] Kurt M., Saban K.O., Kaynak Y., Atakok G., Girit O. (2009). Experimental investigation of plastic injection molding: Assessment of the effects of cavity pressure and mold temperature on the quality of the final products. Mater. Des..

[11] Wang J., Xie P., Ding Y., Yang W. (2009). On-line testing equipment of P-V-T properties of polymers based on an injection molding machine. Polym. Test..

[12] Xie P.C., Wang X.H., Wu T., Ding Y.M., Yang W.M. (2014). Study on packing phase control based on the cavity pressure-temperature during injection molding. Inter. Polym. Process..

[13] Wang J., Mao Q. (2013). A novel process control methodology based on the PVT behavior of Polymer for injection molding. Adv. Polym. Technol..

[14] Gao R.X., Tang X., Gordon G., Kazmer D.O. (2014). Online product quality monitoring through in-process measurement. CIRP Annals—Manuf. Technol..

[15] Gordon G., Kazmer D.O., Tang X., Fan Z., Gao R.X. (2015). Quality control using a multivariate injection molding sensor. Inter. J. Adv. Manuf. Technol..

[16] Chen S.C., Chiu M.C., Tseng Y.L., Tang C.C. Evaluating the through-plane conductivity of molded parts via magnetic field in the injection molding process. Proceedings of the SPE ANTEC Conference.

[17] Lin C.C., Wang W.T., Kuo C.C., Wuet C.L. (2014). Experimental and theoretical study of melt viscosity in injection process. Inter. J. Mech. Mecha. Eng..

[18] Zhao P., Zhou H., He Y., Cai K., Fu J. (2014). A nondestructive online method for monitoring the injection molding process by collecting and analyzing machine running data. Int. J. Adv. Manuf. Technol..

[19] Gefran Co. GE1029 Tie-Bar Strain Sensor. https://www.gefran.com/en/products/424-ge1029-tie-bar-strain-sensor.

[20] Huang M.S., Nian S.C., Chen J.Y., Lin C.Y. (2018). Influence of clamping force on tie-bar elongation, mold separation, and part dimensions in injection molding. Precis. Eng..

[21] Huang M.S., Lin T.Y. (2008). Simulation of a regression-model & PCA based searching method developed for setting the robust injection molding parameters of multi-quality characteristics. Int. J. Heat Mass Transf..

